# A Combined Measurement Method for Large-Size Aerospace Components

**DOI:** 10.3390/s20174843

**Published:** 2020-08-27

**Authors:** Zhilong Zhou, Wei Liu, Qiong Wu, Yuxin Wang, Binchao Yu, Yi Yue, Jiabo Zhang

**Affiliations:** 1 Key Laboratory for Precision and Non-traditional Machining Technology of the Ministry of Education, Dalian University of Technology, Dalian 116024, China; zzl666@mail.dlut.edu.cn (Z.Z.); bzvntsl@mail.dlut.edu.cn (Q.W.); wangyuxinn@mail.dlut.edu.cn (Y.W.); yubinchao@mail.dlut.edu.cn (B.Y.); 2 Beijing Spacecrafts, China Academy of Space Technology, Beijing 100094, China; yuebuaa@sina.com (Y.Y.); airforce_81@163.com (J.Z.)

**Keywords:** combined measurement method, large-size aerospace component, key local features, extrinsic parameter calibration, angular constraint

## Abstract

Automated and high-accuracy three-dimensional (3D) shape measurement is required in quality control of large-size components for the aerospace industry. To eliminate the contradiction between global measurement and local precision measurement control in 3D digitalization for the key local features of the large-size components, a combined measurement method is proposed, including a 3D scanner, a laser tracker, and an industrial robot used as an orienting device, to achieve high-accuracy measurement. As for improving the overall measurement accuracy, an accurate calibration method based on coordinate optimization of common points (COCP) and coordinate optimization of global control points (COGP) is proposed to determine the coordinate systems. Firstly, a coordinate optimization method of common points (COCP) is recommended. Then, a coordinate optimization method of global control points (COGP) based on the angular constraint is proposed for minimizing the measurement errors and improving the measurement accuracy of the position and orientation of the 3D scanner. Finally, a combined measurement system is established, and validation experiments are carried out in laboratory within a distance of 4 m. The calibration experiment results demonstrate that the max and mean errors of the coordinate transformation have been reduced from 0.037 and 0.022 mm to 0.021 and 0.0122 mm. Additionally, the measurement experiment results also show that the combined measurement system features high accuracy.

## 1. Introduction

Large-size components with a large number of supports are commonly seen in modern advanced manufacturing, especially in the aerospace industry. The machining quality of the key local features on the support mounting surface directly impacts the quality of assemblies between the large-scale component and external instruments. On-line machining is necessary because high-precision and high-efficiency machining cannot presently be achieved through off-line processing. Therefore, to ensure on-line machining accuracy, it is essential to measure the key local features on-line at the product design coordinates. The key 3D and geometrical information derived from the measurement includes the component’s position and orientation, the 3D shape, and the location of the key local features on the supports. Furthermore, the measuring range (3 × 3 m to 5 × 6 m) is large, which makes it challenging to locate the support with high accuracy and simultaneously acquire 3D information of the key local feature (25 × 25 mm) with high accuracy (±0.035 mm). The requirement of measuring the key local features in large-scale on-site machining usually cannot be met by the employment of a single measuring device. Additionally, all information needs to be obtained at the same time and transformed into a unified coordinate system, which also makes the large-scale 3D measurement extremely challenging.

Various methods and systems have been proposed for the large-scale 3D measurements [1,2]. The three-coordinate measuring machines (CMMs) have been extensively applied in 3D measurement due to their high accuracy and excellent stability. With the development of noncontact optical measuring equipment and computer vision techniques, visual sensors have been integrated with traditional CMMs [3,4,5]. However, due to the limitation of CMM measurement efficiency, this allows only a small percentage of products to be sampled and inspected. Furthermore, CMM is less used in on-site and on-line measurement due to its structure. When 3D shape information of large-sized components needs to be measured, the 3D shape measurement system integrated with photogrammetry and fringe projection [6] is widely used. However, reflective markers on the target must be attached before the measurement, which interferes with the morphology of the measured part and affects the measurement efficiency. Recently, industrial robots have been extensively applied in the manufacturing field as economical and flexible orienting devices. Therefore, an increasing number of visual sensors are being integrated into robots. Laser scanning, a technology for large-scale 3D shape measurement [7,8,9,10], is more available and economical. However, laser scanning only collects data along limited lines for each measurement, which may result in the robot scanning results containing ripples. Thus, the measurements of the key local features remain barriers to obtaining high accuracy. To extend the measuring range of the laser scanner at designated measurement positions, a movement mechanism [11,12] has been integrated into the laser scanning system. In addition, a linear track or a rotary system will be required to be put into use. However, the movement mechanism inevitably gives rise to errors, reducing the measurement accuracy. The movement mechanism should be calibrated to ensure measurement accuracy. Compared with the laser scanning, structured light profilometry [13,14,15,16,17,18,19,20] has been well developed and widely used for scanning the surface of the object rapidly, as well as acquiring a high-density and high-quality point cloud of a region for each measurement. If the calibration process of the visual sensors is well designed and implemented, their measurement accuracy can be guaranteed [21,22]. Furthermore, compared with the line scanning method, structured light profilometry has a much bigger scanned area and thus is more efficient. Due to the large-size geometry of the component and the finite measuring range of a single station, it is difficult to guarantee the overall measurement accuracy.

To further expand the measurement range for measuring large-size objects and guarantee the overall measurement accuracy, more external measurement devices are being integrated into 3D shape measurement systems [23,24,25,26], such as indoor GPS (iGPS) systems, total stations, and laser trackers. Du, F. et al. [23] developed a large-scale 3D measurement system that combines iGPS, a robot, and a portable scanner. However, the overall measurement accuracy is limited by the measurement property of the iGPS. Paoli, A. et al. [24] developed a 3D measurement system that combines a 3D scanner, a total station, and a robot for automating the measurement process of hull yacht shapes. Several optical target corner cube reflectors are mounted on the robotic system basis and tracked by a total station. However, the robot positioning error is inevitably introduced, and the measurement accuracy is restricted by the robot positioning accuracy. Leica developed a large-scale 3D shape measurement system [25] by combining a laser tracker, T-scan, and a robot. However, it was too expensive to be widely adopted. Du, H. et al. [26] proposed a robot-integrated 3D scanning system, which combines a 3D scanner, a laser tracker, and a robot. The scanner is carried by the robot heading to the planned measurement position during operation. Its end coordinate system is created by rotating the 3D scanner, which is tracked by the laser tracker. However, the laser tracker cannot detect and control the measurement errors during the measurement process.

As the requirements for accuracy have continued to increase, the current measurement methods and systems mentioned above cannot meet the present requirements for high-accuracy on-line measurement of key local features. Besides, the study of the error control in measurement systems is limited. Therefore, a combined measurement method for the large-scale 3D shape measurement of key local features is proposed, combining a 3D scanner, a laser tracker, and an industrial robot. On the basis, a novel calibration method is carried out.

The remainder of the paper is structured as follows: Section 2 introduces the combined measurement method in detail. The calibration of the measurement system is described in Section 3. In Section 4, the proposed method is verified through calibration experiments and measurement experiments, and concluding remarks are provided in Section 5.

## 2. Measurement Principle

### 2.1. Combined Measurement Method

**Figure 1:**
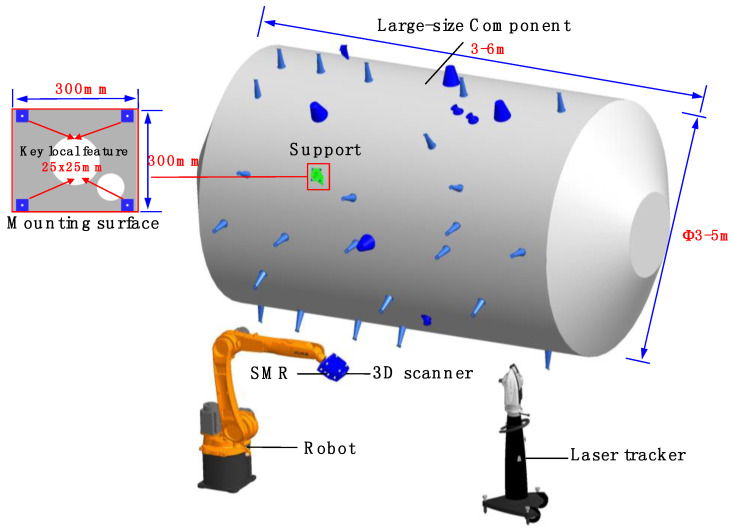
Hardware architecture and the combined measurement scheme.

The proposed combined measurement system mainly incorporates a laser tracker, a 3D scanner, and a robot, as shown in Figure 1. Based on the fringe projection technique, the 3D scanner can capture local high-accuracy 3D shape information. The global measurement method adopts laser trackers to ensure the unity of overall and local measurement accuracy. Additionally, the industrial robot is introduced as the orienting device to improve the efficiency. The 3D scanner will be carried by the robot heading to the discrete measured regions. Then, the laser tracker measures the spherically mounted reflectors (SMRs) rigidly mounted on the base of the 3D scanner. In this way, the position and orientation of the 3D scanner can be acquired. Besides, the acquired data are unified in the world coordinate system defined by the laser tracker.

A calibration method is proposed to improve the overall measurement accuracy of the proposed system. The relationship between the intermediate coordinate system set on the 3D scanner and the 3D scanner measurement coordinate system is deduced by the method. As for improving the overall measurement accuracy, an accurate calibration method based on coordinate optimization of common points (COCP) and coordinate optimization of global control points (COGP) is proposed to determine the coordinate systems. In the calibration process, both the 3D scanner and the laser tracker are used at different positions for measuring the common points and acquiring redundant data. Firstly, the COCP is recommended. Then, the 3D data of the target are obtained by moving the multiview 3D scanner. Meanwhile, the position and orientation of the 3D scanner at each position are acquired by the laser tracker. Moreover, the COGP based on the angular constraint is proposed for controlling the measurement errors and improving the measurement accuracy for the position and orientation of the 3D scanner.

### 2.2. Measurement Model

**Figure 2:**
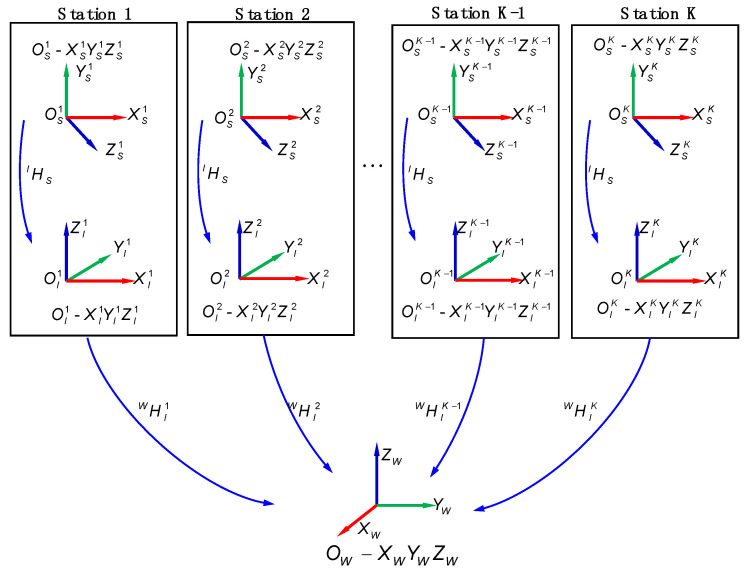
Schematic of the combined measurement model.

The coordinate systems of the proposed system comprise the 3D scanner measurement coordinate system $O_{S}-X_{S}Y_{S}Z_{S}$ (SCS), the intermediate coordinate system $O_{I}-X_{I}Y_{I}Z_{I}$ (ICS), and world coordinate system $O_{W}-X_{W}Y_{W}Z_{W}$ (WCS). To provide laser trackers with the position and orientation of the 3D scanner, ICS is created on the 3D scanner framework and serves as a fixed reference frame of the SCS. Besides, to get the complete data for the key local features on the support mounting surface, the measurement should be implemented multiple times at different stations. Then, the acquired multiview data can be converted into WCS. The schematic of the combined measurement model is shown in Figure 2.

With P being a visual point on the workspace, the coordinate mapping model between WCS and SCS is expressed as follows:

(1) PW=HIWHSIPs

where $P_{W}$ and $P_{S}$ are the homogeneous coordinates of the visual point *P* in WCS and SCS, respectively. HIW and HSI are 4×4 homogeneous transformation matrices. HIW denotes the transformation relationship between WCS and ICS while HSI, as an invariable during the measurement process, reflects the coordinate transformation relationship between ICS and SCS. As the calibration of transformation for the ICS and the SCS is viewed as extrinsic parameter calibration, an accurate calibration method will be introduced in the following section.

## 3. Calibration of the Measurement System

The high-precision model for coordinate transformation between SCS and ICS is established by extrinsic parameter calibration. Additionally, the extrinsic parameter calibration has to be performed before the combined measurement system is applied for large-scale metrology, and there is no calibration procedure during the measurement process. Therefore, an accurate extrinsic parameter calibration result is a critical factor in ensuring the overall measurement accuracy of the proposed system.

As for improving the overall measurement accuracy and minimizing the measurement errors, a extrinsic parameter calibration method based on COCP and COGP optimization is proposed. Firstly, the COCP is recommended. Then, the COGP based on the angular constraint is proposed for minimizing the measurement errors and improving the measurement accuracy of the position and orientation of the 3D scanner.

### 3.1. Calibration Principle

The homogeneous coordinates of the points $P_{i}$ in two coordinate systems can be denoted as $P_{i1}=(x_{pi1},y_{pi1},z_{pi1},1)$ and $P_{i2}=(x_{pi2},y_{pi2},z_{pi2},1)$. The relationship can be expressed as follows:

(2) $$\begin{array}{c} P_{i1}=HP_{i2},i=1,2,\cdots ,n\\ H=\left[\begin{array}{cc} R_{3\times 3}(\alpha \;\beta \;\gamma ) & T_{3\times 1}(\begin{array}{ccc} T_{x} & T_{y} & T_{z} \end{array})\\ 0 & 1 \end{array}\right]\\ R_{3\times 3}(\alpha \;\beta \;\gamma )=\left[\begin{array}{ccc} \cos\alpha \cos\beta & \cos\alpha \sin\beta \sin\gamma -\sin\alpha \cos\gamma & \cos\alpha \sin\beta \cos\gamma +\sin\alpha \sin\gamma \\ \sin\alpha \cos\beta & \sin\alpha \sin\beta \sin\gamma +\cos\alpha \cos\gamma & \sin\alpha \sin\beta \cos\gamma -\cos\alpha \sin\gamma \\ -\sin\beta & \cos\beta \sin\gamma & \cos\beta \cos\gamma \end{array}\right] \end{array}$$

where H is the homogeneous transformation matrix. R(α β γ) is a rotation matrix, and α,β,γ are the angles of Cardan. T is a translation vector.

**Figure 3:**
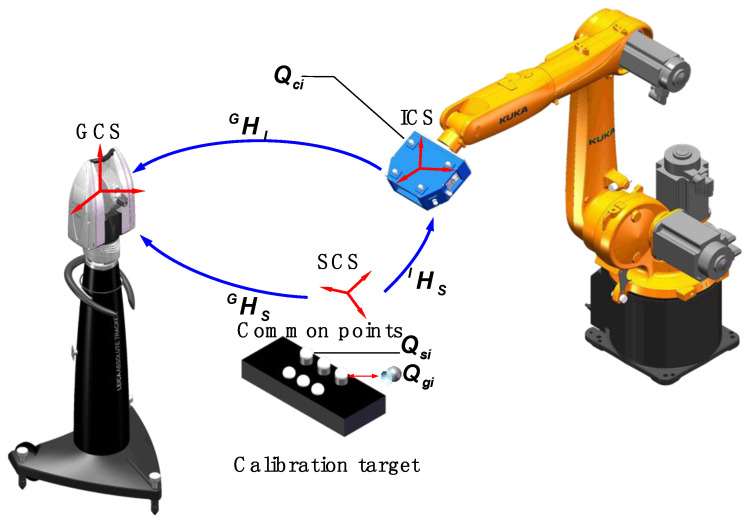
Principle of extrinsic parameter calibration.

The combined calibration system consists of a 3D scanner, a laser tracker, an industrial robot, and the calibration target. The principle of extrinsic parameter calibration is shown in Figure 3. Firstly, to establish the transformation relationship HSG between SCS and GCS, the laser tracker and the 3D scanner measure the common points arranged on the calibration target. Then, the laser tracker can locate and track the position and orientation of the 3D scanner by measuring the coordinates of the global control points, and the transformation matrix HIG is established after the process. Finally, according to the two transformation matrixes above, the extrinsic parameter matrix HSI can be calculated.

Four or more noncollinear SMRs set on the 3D scanner, known as global control points, are denoted as $Q_{ci},ci=1,2,\cdots ,cn$. Besides, the homogeneous coordinates of $Q_{ci}$ can be denoted as $(X_{ci}^{I},Y_{ci}^{I},Z_{ci}^{I},1)$ in ICS. Two groups of target observation points replaced by each other are arranged on the calibration target for ensuring the accuracy of extrinsic parameter calibration. As the standard ceramic spheres take the place of SMRs, the sphere centers of the SMRs are basically in the same positions as those of sphere centers of the standard ceramic spheres. The homogeneous coordinates of the laser tracker observation points $Q_{gi},gi=1,2,\cdots ,gn$ can be denoted as $P_{gi}=(X_{gi}^{G},Y_{gi}^{G},Z_{gi}^{G},1)$ in laser tracker measurement coordinate system $O_{G}-X_{G}Y_{G}Z_{G}$ (GCS), and the homogeneous coordinates of the 3D scanner observation points $Q_{si},si=1,2,\cdots ,sn$ can be denoted as $P_{si}=(X_{si}^{S},Y_{si}^{S},Z_{si}^{S},1)$ in SCS; the following relationship between them exists:

(3) Pgi=HSGPsi

The homogeneous coordinates of $Q_{ci}$ can be denoted as $P_{ci}=(X_{ci}^{G},Y_{ci}^{G},Z_{ci}^{G},1)$ in GCS, and then the following relationship between $Q_{ci}$ in ICS and $P_{ci}$ in GCS is expressed as follows:

(4) Pci=HIGQci

where HIG is the transformation matrix between GCS and ICS.

According to Equations (2)–(4), the HSI can be calculated as follows:

(5) HSI=HI−1GHSG

Improving the accuracy of transformation matrixes HSG and HIG is the key to improving the accuracy of transformation matrix HSI. However, the measurement errors of the laser tracker and the 3D scanner could lead to transformation parameter errors. Therefore, to improve the accuracy of extrinsic parameter calibration by minimizing measurement errors, the coordinate optimization method of the common points is proposed in Section 3.2, and the coordinate optimization method of the global control points is proposed in Section 3.3.

### 3.2. Optimization of the Coordinates of Common Points

The common points arranged on the calibration target are measured by both the laser tracker and the 3D scanner. COCP is proposed to minimize measurement errors. In addition, it optimizes the transformation parameters of the HSG, which consists of three angle parameters in matrix RSG(α β γ) and three translation parameters in TSG(TxTyTz).

**Figure 4:**
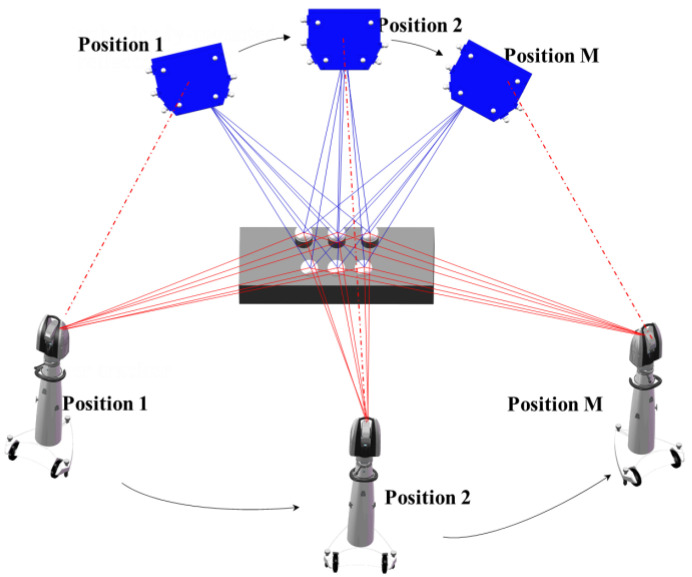
Schematic of the adjustment optimization.

Figure 4 shows common points that are measured at M positions. If the coordinate system of the first position is taken as the reference coordinate system, the Cartesian coordinates of the common points obtained by the laser tracker and the 3D scanner at first position can be denoted as $P_{gi}^{G0}=(X_{gi}^{G0},Y_{gi}^{G0},Z_{gi}^{G0})$ and $P_{si}^{S0}=(X_{si}^{S0},Y_{si}^{S0},Z_{si}^{S0})$, respectively. The Cartesian coordinates of the common points measured in other positions can be denoted as $p_{gi}^{Gm}=(x_{gi}^{Gm},y_{gi}^{Gm},z_{gi}^{Gm}),m=1,\cdots ,M-1;gi=1,\cdots ,gn$ and $p_{si}^{Sm}=(x_{si}^{Sm},y_{si}^{Sm},z_{si}^{Sm}),m=1,\cdots ,M-1;si=1,\cdots ,sn$. If measurement errors are considered, Equation (2) can be rewritten as follows:

(6) $$\{\begin{array}{c} P_{gi}^{G0}+\Delta P_{gi}^{G0m}=R_{gi}^{Gm}p_{gi}^{Gm}+T_{gi}^{Gm}\\ P_{si}^{S0}+\Delta P_{si}^{S0m}=R_{si}^{Sm}p_{si}^{Sm}+T_{si}^{Sm} \end{array}$$

where $\Delta P_{gi}^{G0m}=(\Delta X_{gi}^{G0m},\Delta Y_{gi}^{G0m},\Delta Z_{gi}^{G0m})$ and $\Delta P_{si}^{S0m}=(\Delta X_{si}^{S0m},\Delta Y_{si}^{S0m},\Delta Z_{si}^{S0m})$ represent the correction values for common points of the laser tracker and the 3D scanner, respectively. The simultaneous equation of measurement errors is as follows:

(7) $$\begin{array}{l} (\Delta X_{gi}^{G0m},\Delta Y_{gi}^{G0m},\Delta Z_{gi}^{G0m},\cdots ,\Delta X_{si}^{S0m},\Delta Y_{si}^{S0m},\Delta Z_{si}^{S0m})=\\ \underset{\left(\Delta X_{gi}^{G0m},\cdots ,\Delta Z_{si}^{S0m}\right)}{\mathrm{argmin}}\left\{\sum_{m=1}^{M}\left[\sum_{si=1}^{sn}{\left(P_{gi}^{G0}-R_{gi}^{Gm}p_{gi}^{Gm}-T_{gi}^{Gm}\right)}^{2}+\sum_{gi=1}^{gn}{\left(P_{si}^{S0}-R_{si}^{Sm}p_{si}^{Sm}-T_{si}^{Sm}\right)}^{2}\right]\right\} \end{array}$$

The rotation and translation matrix between the laser tracker and 3D scanner at all positions can be computed by the Procrustes method [27]. Based on this, the correction value of coordinates of the common points can be calculated by the rank-defect network adjustment algorithm [28]. As a result, the coordinate values of the common points are optimized. Thereby, the accuracy of the transformation parameters of HSG is improved.

### 3.3. Optimization of the Coordinates of Global Control Points

**Figure 5:**
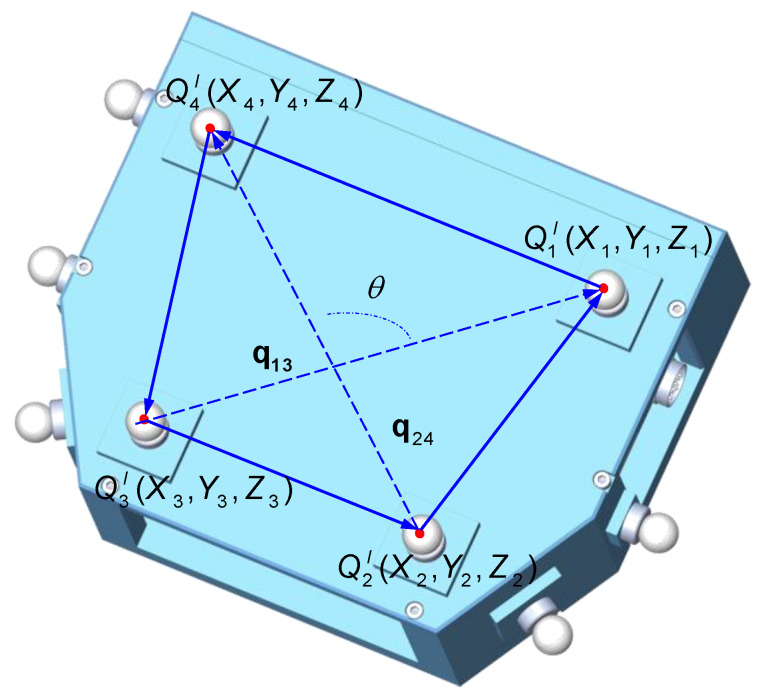
Schematic of the spatial angle.

Due to environmental uncertainties and instrument instability, errors in the measurement of global control points are unavoidable. To solve the problem of unknown and uncontrollable error in measuring the global control points and optimize the transformation parameters of HIG, COGP based on the angular constraint is proposed to obtain the correction values of coordinates of the global control points. Because the angle between two vectors in Euclidean space is independent of the coordinate system [29], the geometric information of the global control points set on the 3D scanner is fully used. In four global control points, a vector is established by two target points, and the angle between the vectors $q_{13}$ and $q_{24}$ is θ, as shown in Figure 5; the four target points can make up six vectors, which can form 15 angles.

CMM is used to calibrate the angle values, which are set as the nominal angles. We can obtain the angle error equation by calculating the difference between the actual angle values measured from the on-site measurement and the nominal angle values. Then, the normal equation is obtained by the least squares method.

The method for finding the angle between two nonzero vectors is expressed as follows:

(8) $$\cos\theta =\frac{q_{13}\cdot q_{24}}{\left\|q_{13}\right\|\left\|q_{24}\right\|}$$

Therefore, the angle θ could be obtained by the arc cosine function as follows:

(9) $$\theta =\arccos(\frac{\left(x_{3}-x_{1})(x_{4}-x_{2})+(y_{3}-y_{1})(y_{4}-y_{2})+(z_{3}-z_{1})(z_{4}-z_{1}\right)}{\sqrt{{\left(x_{3}-x_{1}\right)}^{2}+{\left(y_{3}-y_{1}\right)}^{2}+{\left(z_{3}-z_{1}\right)}^{2}}\sqrt{{\left(x_{4}-x_{2}\right)}^{2}+{\left(y_{4}-y_{2}\right)}^{2}+{\left(z_{4}-z_{2}\right)}^{2}}})$$

Equation (9) is expanded by Taylor’s formula, and the second-order term is ignored. Therefore, the linearized equation of angular constraint is expressed as follows:

(10) $$f(\Delta {\overset{⌢}{x}}_{i},\Delta {\overset{⌢}{y}}_{i},\Delta {\overset{⌢}{z}}_{i})={\hat{\theta }}_{i}-\theta_{i}^{0}-(\frac{\partial \theta_{i}}{\partial x_{1}}\Delta {\overset{⌢}{x}}_{1}+\frac{\partial \theta_{i}}{\partial y_{1}}\Delta {\overset{⌢}{y}}_{1}+\frac{\partial \theta_{i}}{\partial z_{1}}\Delta {\overset{⌢}{z}}_{1}+\cdots +\frac{\partial \theta_{i}}{\partial z_{n}}\Delta {\overset{⌢}{z}}_{n}),i=1,2,\cdots ,15;n=1,2,\cdots 4$$

where $\Delta {\overset{⌢}{x}}_{i},\Delta {\overset{⌢}{y}}_{i},\Delta {\overset{⌢}{z}}_{i}$ are the optimized correction values of coordinates of the global control points, and $\Delta \overset{⌢}{X}=[\Delta {\overset{⌢}{x}}_{1}\;\Delta {\overset{⌢}{y}}_{1}\;\Delta {\overset{⌢}{z}}_{1}\;\Delta {\overset{⌢}{x}}_{2}\;\Delta {\overset{⌢}{y}}_{2}\;\Delta {\overset{⌢}{z}}_{2}\cdots \Delta {\overset{⌢}{x}}_{n}\;\Delta {\overset{⌢}{y}}_{n}\;\Delta {\overset{⌢}{z}}_{n}]$ is a vector of the correction values.

The angle error equation is expressed as follows:

(11) $$w_{i}={\hat{\theta }}_{i}-\theta_{i}^{0}$$

where ${\hat{\theta }}_{i}$ are nominal angles, and $\theta_{0}$ are actual angles.

In an alternative way, Equation (10) can be rewritten as follows:

(12) $$W=\left[\begin{array}{cccccc} \frac{\partial \theta_{1}}{\partial x_{1}} & \frac{\partial \theta_{1}}{\partial y_{1}} & \frac{\partial \theta_{1}}{\partial z_{1}} & \frac{\partial \theta_{1}}{\partial x_{2}} & \cdots & \frac{\partial \theta_{1}}{\partial z_{n}}\\ \frac{\partial \theta_{2}}{\partial x_{1}} & \frac{\partial \theta_{2}}{\partial y_{1}} & \frac{\partial \theta_{2}}{\partial z_{1}} & \frac{\partial \theta_{2}}{\partial x_{2}} & \cdots & \frac{\partial \theta_{2}}{\partial z_{n}}\\ \vdots & \vdots & \vdots & \vdots & \cdots & \vdots \\ \frac{\partial \theta_{i}}{\partial x_{1}} & \frac{\partial \theta_{i}}{\partial y_{1}} & \frac{\partial \theta_{i}}{\partial z_{1}} & \frac{\partial \theta_{i}}{\partial x_{2}} & \cdots & \frac{\partial \theta_{i}}{\partial z_{n}} \end{array}\right]\left[\begin{array}{c} \Delta {\overset{⌢}{x}}_{1}\\ \Delta {\overset{⌢}{y}}_{1}\\ \Delta {\overset{⌢}{z}}_{1}\\ \Delta {\overset{⌢}{x}}_{2}\\ \vdots \\ \Delta {\overset{⌢}{y}}_{n}\\ \Delta {\overset{⌢}{z}}_{n} \end{array}\right]-\left[\begin{array}{c} w_{1}\\ w_{2}\\ w_{3}\\ \vdots \\ w_{i} \end{array}\right]=B\Delta \overset{⌢}{X}-d$$

where $d={\hat{\theta }}_{i}-\theta_{i}^{0}$ is the angle error vector.

Then, the normal equation is as follows:

(13) $$(B^{T}UB)\Delta \overset{⌢}{X}=B^{T}Ud$$

where U is the weight matrix.

The objective function for finding the best coordinate estimates can be expressed as follows:

(14) $$\left(\Delta {\overset{⌢}{x}}_{1},\Delta {\overset{⌢}{y}}_{1},\Delta {\overset{⌢}{z}}_{1},\cdots ,\Delta {\overset{⌢}{x}}_{n},\Delta {\overset{⌢}{y}}_{n},\Delta {\overset{⌢}{z}}_{n}\right)=\underset{\left(\Delta {\overset{⌢}{x}}_{1},\cdots ,\Delta {\overset{⌢}{z}}_{n}\right)}{\arg\;\min}\left[U{\left\|B\Delta \overset{⌢}{X}-d\right\|}_{2}^{2}+U{\left\|\Delta \overset{⌢}{X}\right\|}_{2}^{2}\right]$$

The angle adjustment is conducted to obtain the optimal estimate value by solving the error matrix equation in the least squares norm. However, the traditional least squares method requires the coefficient matrix to be a nonsingular or full rank matrix. The coefficient matrix $B^{T}UB$ in Equation (13) is an ill-conditioned matrix with maximum condition number $cond(B^{T}UB)=\lambda_{\max}/\lambda_{\min}$ ($\lambda_{\max}$ and $\lambda_{\min}$ represent the maximum and minimum eigenvalues of coefficient matrix $B^{T}UB$), and the result of this solution is extremely unstable.

To obtain the optimal solution, a two-objective optimization formula can be constructed as follows:

(15) $$Q(\Delta \overset{⌢}{X})=\min(\left\|BU\Delta \overset{⌢}{X}-d\right\|,\left\|\Delta \overset{⌢}{X}\right\|)$$

According to Tikhonov’s regularization method, the objective function of Equation (15) based on the ridge estimation algorithm is given as follows:

(16) $$Q(\Delta \overset{⌢}{X})={\left\|BU\Delta \overset{⌢}{X}-d\right\|}_{2}^{2}+\alpha {\left\|\Delta \overset{⌢}{X}\right\|}_{2}^{2}=\Delta {\overset{⌢}{X}}^{T}(B^{T}UB+\alpha I)\Delta \overset{⌢}{X}-2d^{T}BU\Delta \overset{⌢}{X}+d^{T}d=\min$$

where the non-negative parameter α is the ridge estimation parameter, and I is the unit matrix. Finding the conjugate gradient of $\Delta \overset{⌢}{X}$ in Equation (16), we have

(17) $$\frac{\partial Q(\Delta \overset{⌢}{X})}{\partial \Delta {\overset{⌢}{X}}^{T}}=(B^{T}UB+\alpha I)\Delta \overset{⌢}{X}-BU^{T}d$$

According to the extremum condition, let Equation (17) be equal to 0. Therefore, the final solution can be expressed as follows:

(18) $$\Delta \overset{⌢}{X}={\left(B^{T}UB+\alpha I\right)}^{-1}B^{T}Ud$$

where the damping term αI added to the main diagonal of the coefficient matrix $B^{T}UB$ in Equation (18) can overcome the ill-conditioned effect of the coefficient matrix. Thus, a stable solution can be obtained.

The ridge estimation method can change singular matrix $B^{T}UB$ into a nonsingular matrix. Besides, it ensures the stability for the solution of the ill-conditioned equation. The appropriate ridge parameter α can be solved by the L-curve method [30], which can reduce the condition number of the equation and change the ill-conditioned equation into a well-conditioned equation. As a result, the coordinate values of the global control points are optimized. Thereby, the accuracy of the transformation parameters of HIG is improved.

## 4. Experiments and Discussion

**Figure 6:**
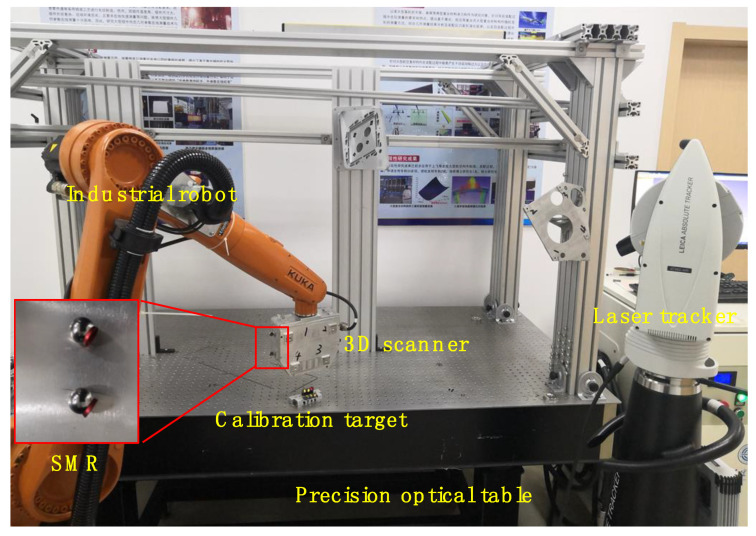
Overview of the experimental setup of the system.

Based on the principle detailed in Section 3.1, the extrinsic parameter calibration was carried out. The calibration method mentioned in Section 3 can be verified through calibration experiments and measurement experiments. The experimental setup of the combined measurement system is shown in Figure 6. The 3D scanner was mounted on the 6-DOF industrial robot KR10R1420 manufactured by KUKA Corporation, and several SMRs set on the 3D scanner ensure that the position and orientation of the 3D scanner can be tracked by the laser tracker. The laser tracker was the Leica AT960, with an accuracy of 0.015 mm ± 0.006 mm/m, which can be connected to the computer by Gigabit Ethernet (GBE). The off-the-shelf visual sensor is a binocular structured light scanner with precision of 0.012 mm, resolution up to 0.020 mm, and scan range of 30 × 40 × 25 mm.

**Figure 7:**
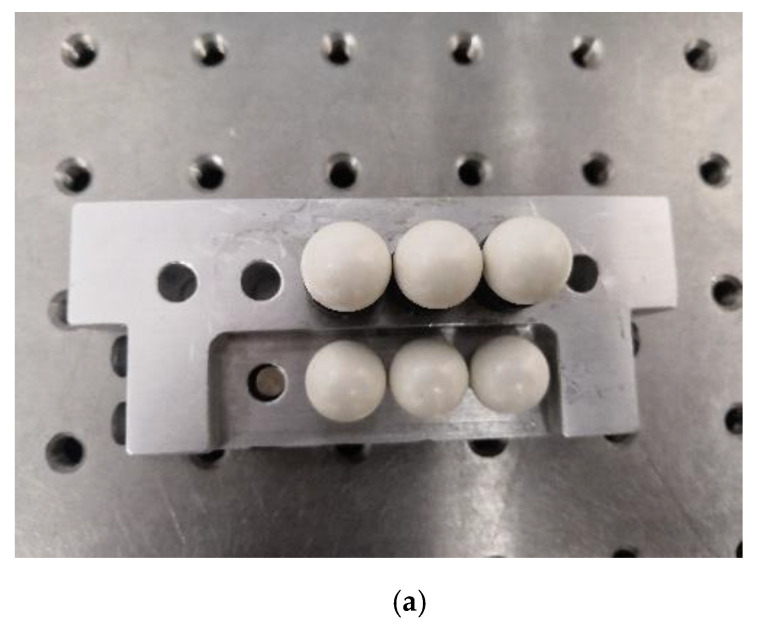

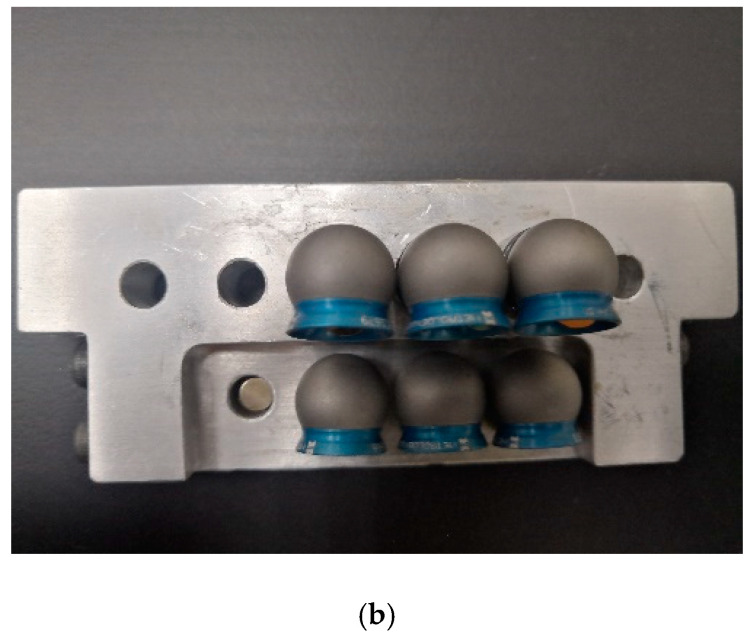
Calibration target: (**a**) standard ceramic spheres; (**b**) spherically mounted reflectors.

Figure 7 shows a specific calibration target designed for extrinsic parameter calibration and experiment purposes. Six sophisticated magnetic nests (SMNs) for the SMRs of laser tracker and the standard ceramic spheres of the 3D scanner were rigidly assembled on the aluminum plate. The standard ceramic spheres and the SMRs were first inspected on a CMM, and the maximum deviation of the diameter of the spheres was found to be about 0.003 mm. Therefore, supposing that the standard ceramic spheres take the place of the SMRs, the SMR center position would be aligned with that of the standard sphere center. All experiments were performed in a stable laboratory environment. The temperature varied between 22 and 23 °C, and the relative humidity varied between 55 and 60%.

### 4.1. Incidence Angle Experiment

**Figure 8:**
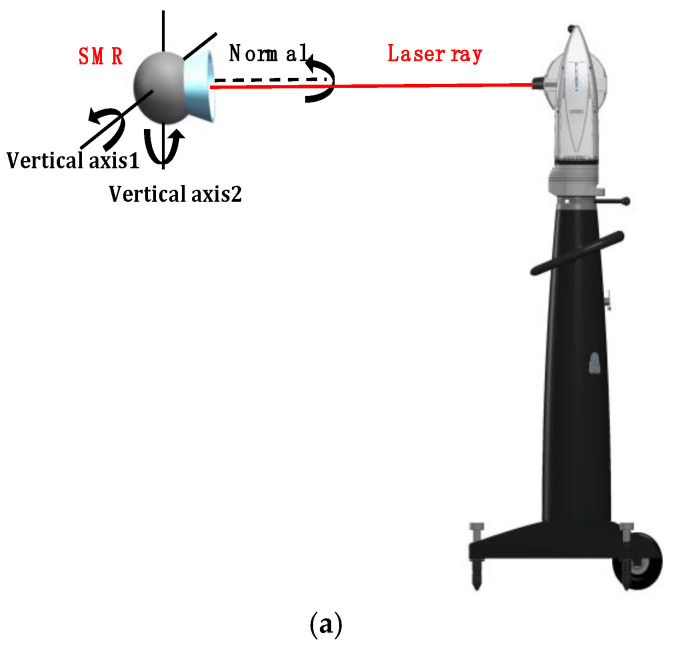

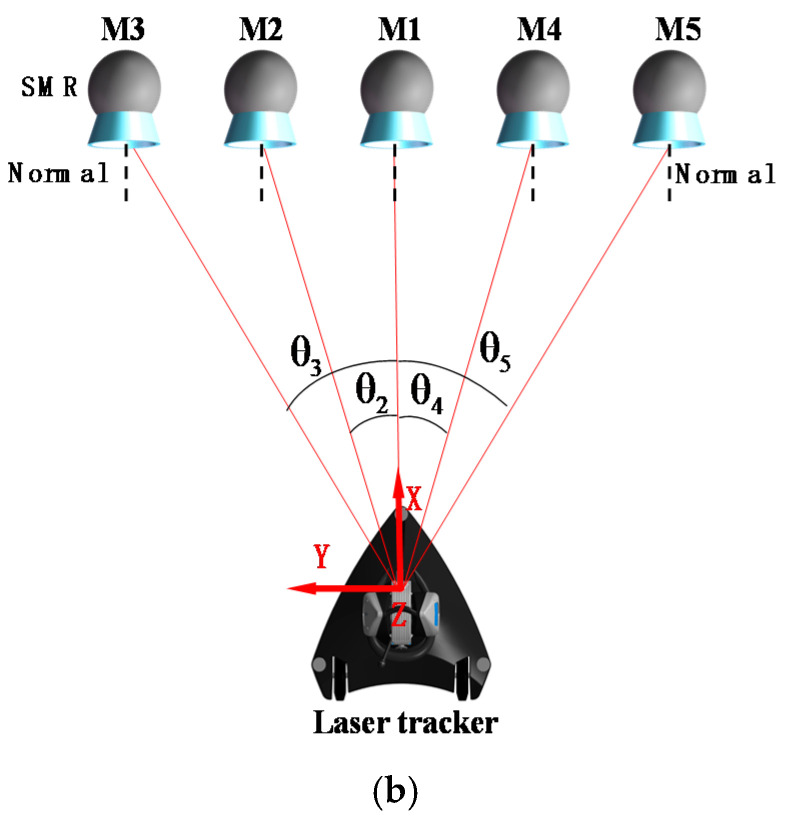
Schematic of the incident angle of the laser ray: (**a**) incident angle of SMR; (**b**) incident angle of laser tracker.

To minimize measurement errors, the influence of the incident angle error of the laser ray on the measurement accuracy of the laser tracker was analyzed under different circumstances. Figure 8a shows the SMR installed at the same height as the laser tracker. Rotating the SMR around the normal, vertical axis 1, and vertical axis 2, we collected 100 measurements for each operation to obtain the average value. The measurement results in Table 1 show that the maximum error can reach 0.008 mm with the change of SMR pose. Figure 8b also shows the SMR installed at the same height as the laser tracker. In the laser tracker measurement coordinate system, the five measuring points were distributed in a straight line and parallel to the XOY plane. The average value was also obtained by measuring 100 times within 2 m. The results of the analysis are shown in Table 2.

In Table 2, the standard deviation of the X-coordinate value $\sigma_{X}$ is close to the standard deviation of the length measurement result $\sigma_{r}$. However, $\sigma_{Y}$ (the standard deviation of the Y-coordinate value) and $\sigma_{Z}$ (the standard deviation of the Z-coordinate value) are relatively large. Because the angle measurement accuracy of the laser tracker is relatively low, larger angles result in lower measurement accuracy. Angle measurement error is the main factor affecting the measurement accuracy of the laser tracker. Therefore, the optimization methods for reducing the incident angle error can be adopted to minimize the measurement errors during the measurement process.

### 4.2. Calibration Experiment

Figure 6 shows a combined calibration system, which combines a 3D scanner, a laser tracker, an industrial robot, and the calibration target. The common points were measured by the laser tracker and the 3D scanner at eight positions. Firstly, several SMRs were mounted on the SMNs of the calibration target and the 3D scanner, and centers of SMRs were measured by the laser tracker. Then, the 3D scanner measured the standard ceramic spheres mounted on the SMNs of the calibration target. Finally, the optimization data and correction data of common points of the laser tracker and the 3D scanner were obtained according to the method introduced in Section 3.2, as shown in Table 3 and Table 4.

Table 5 shows the optimization data and correction data of global control points obtained according to the method introduced in Section 3.3.

The distance errors between the measurement values and the nominal values can be calculated by using the coordinate values of global control points optimized by COGP optimization method. The results are shown in Table 6. The max deviation and RMS errors were reduced from 0.0179 and 0.0111 mm to 0.0115 and 0.0074 mm. This demonstrates that the measurement accuracy of global control points can be improved by the angle constraint method.

With the optimization data of the global control points, an accurate result of the transformation matrix HIG is given as follows:

HIG=[0.7640310.0577440.6425891265.8035−0.641955−0.03140780.7660991357.82780.064420−0.9978370.013073−874.49200001]

With all data mentioned above, the accurate result of the extrinsic parameter calibration can be calculated, according to Equation (5), as follows:

HSI=[−0.031708−0.998010.05444425.2444030.038536−0.055651−0.99771210.358440.99875−0.0295370.04022439.1091190001]

**Figure 9:**
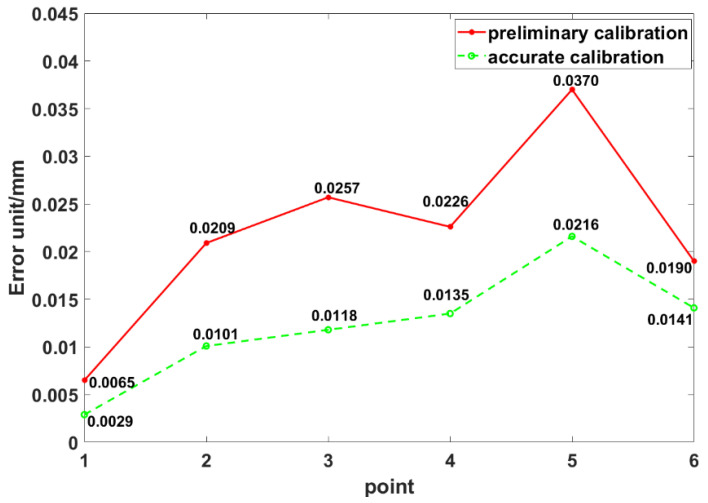
Errors of the coordinate transformation after preliminary and accurate calibration.

As shown in Figure 9, with the accurate calibration result, the max and mean errors of the coordinate transformation were reduced from 0.037 and 0.022 mm to 0.021 and 0.0122 mm. The results demonstrate that the calibration accuracy has been improved significantly, and the calibration method achieves high accuracy.

### 4.3. Measurement Experiment

After the extrinsic parameter calibration, further experiments were needed to verify the performance and the accuracy of the proposed system. Section 4.3.1 detail how the performance of the system was assessed by the support measurement test. The measurement accuracy verification is described in Section 4.3.2.

#### 4.3.1. Support Measurement Test

**Figure 10:**
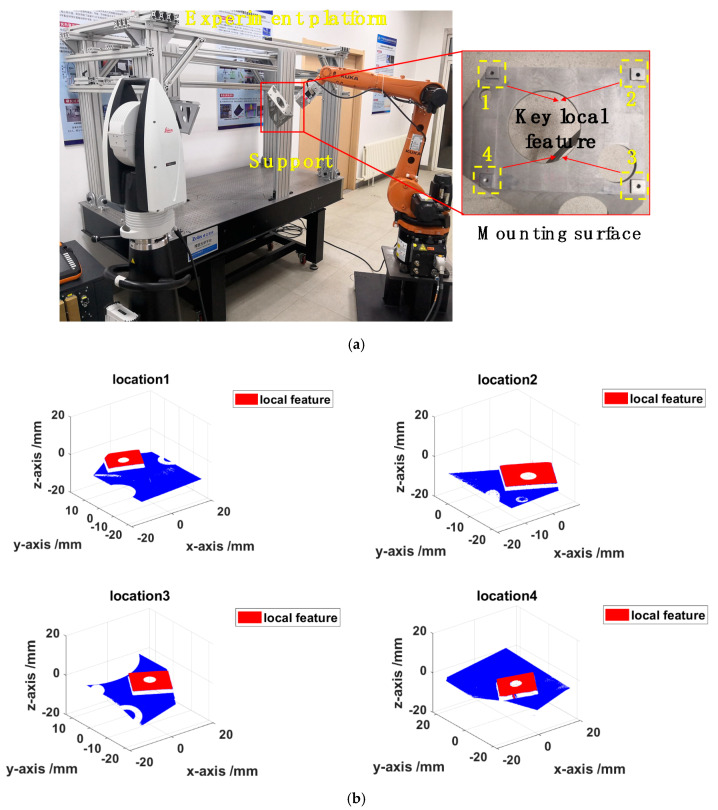

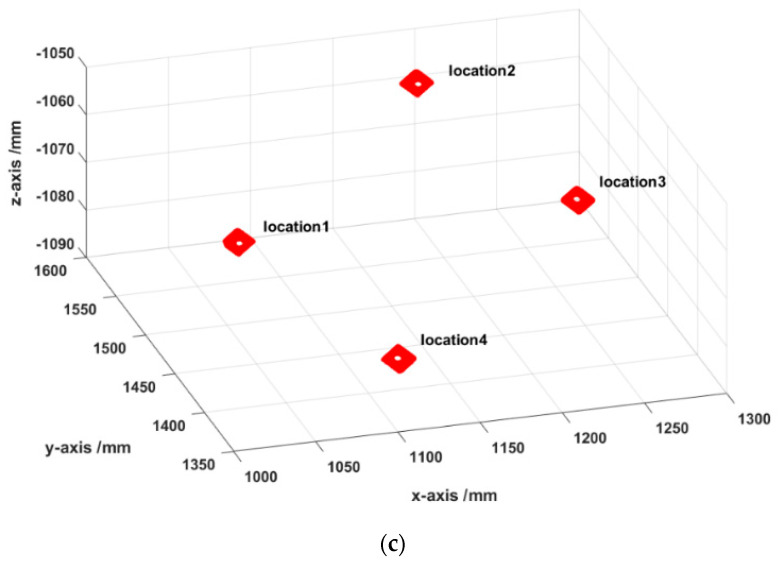
(**a**) Experimental setup for measuring the key local features. (**b**) Surface point clouds from four measurements. (**c**) Reconstructed point clouds of the key local features.

To measure key local features of the support mounting surface with the size of 300 × 300 × 100 mm, the proposed combined measurement system was applied to measure a support fixed on the experiment platform in the laboratory, as shown in Figure 10a. Firstly, the robot moved the 3D scanner to the discrete regions that needed to be measured, and then the 3D scanner scanned the region. Meanwhile, the laser tracker measured the SMRs fixed on the 3D scanner to obtain the position and orientation of 3D scanner. Finally, the 3D information of the local key features could be obtained by the 3D scanner at different robot positions. Figure 10b shows the 3D point clouds obtained at each measurement position. Furthermore, the 3D point clouds of the local key features were preprocessed and then unified into the world frame according to Equation (1), as shown in Figure 10c. This test demonstrated that the combined measurement system can be applied to measure key local features of the support mounting surface.

#### 4.3.2. Accuracy Verification

**Figure 11:**
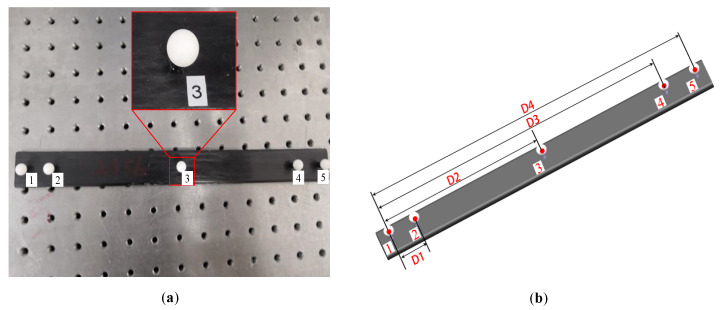
Metric tool for accuracy verification: (**a**) standard ruler; (**b**) distance between the centers of each pair of spheres.

To verify the measurement accuracy of the system, a metric tool that consisted of five homogeneous and standard ceramic spheres with approximate diameter was designed and adopted, as shown in Figure 11. The distance between the centers of two spheres was measured on the CMM, and the results are as follows: D1 = 24.7581 mm, D2 = 149.8429 mm, D3 = 269.7165 mm, and D4 = 299.5461 mm.

The distance errors between the measured values and nominal values were computed on the basis of the model in Equation (1). Table 7 presents the distance errors among the centers of the five spheres, which were measured by the combined measurement system within 4 m. To better demonstrate the errors, the mean value (μ), standard deviation value (σ), and root-mean-square error (RMS error) are summarized in Table 5. The proposed method can be verified by these quantitative statistical results; it results in relatively high accuracy in 3D measurement, with a space length measuring error of less than 0.03%.

## 5. Conclusions

This paper has proposed a combined measurement method for high-accuracy large-scale 3D metrology, where a 3D scanner and a laser tracker are combined to conduct highly accurate measurement of key local features. In this measurement system, the orienting device, an industrial robot, is applied. The principle of measurement has been illustrated in detail. As for improving the overall measurement accuracy, an accurate calibration method based on COCP and COGP optimization has been proposed to determine the coordinate systems. Firstly, the COCP has been recommended. Then, the COGP based on the angular constraint has been proposed for minimizing the measurement errors and improving the measurement accuracy of the position and orientation of the 3D scanner. The calibration experiment results demonstrate that the max and mean errors of the coordinate transformation have been reduced from 0.037 and 0.022 mm to 0.021 and 0.0122 mm. The application of measuring a support has also been performed to demonstrate that the measurement process is simple and efficient. Finally, a metric tool has been developed, which helped to verify the measurement accuracy of the proposed system. Future work will focus on the improvement of measuring accuracy in larger measurement range.

## Figures and Tables

**Table 1:** Errors of the incident angle of the laser ray.

No.	Axis of Rotation	Rotation Angle (°)	X (mm)	Y (mm)	Z (mm)	Distance between Two Points (mm)
1	Normal	0	1863.6033	509.8774	0.2825	0.0036
2	Normal	170–180	1863.6022	509.8751	0.2800	
3	Normal	80–90	1863.6042	509.8732	0.2811	0.0064
4	Normal	260–270	1863.6018	509.8776	0.2771	
5	Vertical axis 1	0–7	1863.6031	509.8772	0.2811	0.0044
6	Vertical axis 1	7–15	1863.6012	509.8740	0.2777	
7	Vertical axis 1	−7 to 0	1863.6032	509.8790	0.2800	0.0051
8	Vertical axis 1	−15 to −7	1863.6011	509.8786	0.2846	
9	Vertical axis 1	5–10	1863.6028	509.8773	0.2815	0.0072
10	Vertical axis 1	20–25	1863.6017	509.8723	0.2773	
11	Vertical axis 2	0–7	1863.6033	509.8777	0.2812	0.0045
12	Vertical axis 2	7–15	1863.6018	509.8790	0.2772	
13	Vertical axis 2	−7 to 0	1863.6038	509.8768	0.2798	0.0052
14	Vertical axis 2	−15 to −7	1863.6014	509.8733	0.2768	
15	Vertical axis 2	−10 to −5	1863.6035	509.8786	0.2812	0.0081
16	Vertical axis 2	−25 to −20	1863.5994	509.8733	0.2768	

**Table 2:** Results of the analysis of the angle measurement of the laser tracker.

No.	$\theta_{i}/{}^{\circ}$	$\sigma_{X}/\mathrm{mm}$	$\sigma_{Y}/\mathrm{mm}$	$\sigma_{Z}/\mathrm{mm}$	$\sigma_{r}/\mathrm{mm}$
M1	0	0.0008	0.0019	0.0017	0.00105
M2	7	0.0016	0.0022	0.0025	0.0013
M3	15	0.0025	0.0064	0.0053	0.0019
M4	−7	0.0012	0.0037	0.0031	0.0014
M5	−15	0.0023	0.0066	0.0054	0.0018

**Table 3:** Optimization data and correction data of common points of the laser tracker (unit: mm).

No.	${\overset{⌢}{X}}_{gi}^{G0}$	${\overset{⌢}{Y}}_{gi}^{G0}$	${\overset{⌢}{Z}}_{gi}^{G0}$	$\Delta X_{gi}^{G01}$	$\Delta Y_{gi}^{G01}$	$\Delta Z_{gi}^{G01}$
1	1321.1788	1375.8569	−1076.7822	0.0071	0.0037	−0.0032
2	1331.0683	1367.4658	−1076.7346	0.0020	−0.0097	−0.0085
3	1340.7795	1358.6840	−1076.6104	0.0087	0.0070	−0.0016
4	1330.4339	1346.7215	−1089.3309	−0.0031	0.0085	0.0010
5	1320.5345	1355.2887	−1089.3385	−0.0162	−0.0092	0.0102
6	1310.7323	1363.7794	−1089.4009	−0.0076	0.0046	−0.0036

**Table 4:** Optimization data and correction data of common points of the 3D scanner (unit: mm).

No.	${\overset{⌢}{X}}_{si}^{S0}$	${\overset{⌢}{Y}}_{si}^{S0}$	${\overset{⌢}{Z}}_{si}^{S0}$	$\Delta X_{si}^{S01}$	$\Delta Y_{si}^{S01}$	$\Delta Z_{si}^{S01}$
1	7.6498	7.6287	5.7611	−0.0003	−0.0007	0.0021
2	7.1858	−5.3400	5.6725	0.0004	0.0010	−0.0015
3	6.3278	−18.4021	5.6586	−0.0002	0.0004	−0.0028
4	−9.1182	−17.5664	−7.4796	−0.0004	0.0004	0.0025
5	−8.5539	−4.5038	−7.3723	0.0007	−0.0013	−0.0019
6	−7.9429	8.4726	−7.2901	0.0005	0.0007	0.0027

**Table 5:** Optimization data and correction data of global control points (unit: mm).

No.	${\overset{⌢}{X}}_{ci}^{G0}$	${\overset{⌢}{Y}}_{ci}^{G0}$	${\overset{⌢}{Z}}_{ci}^{G0}$	$\Delta {\overset{⌢}{x}}_{ci}^{0}$	$\Delta {\overset{⌢}{y}}_{ci}^{0}$	$\Delta {\overset{⌢}{z}}_{ci}^{0}$
1	1265.8035	1357.8278	−874.4920	−0.0059	0.0029	−0.0028
2	1350.6384	1286.5477	−867.3390	0.0008	−0.0013	−0.0052
3	1331.8294	1303.4228	−931.7086	−0.0014	0.0002	−0.0066
4	1241.3520	1375.0563	−934.6902	0.0002	−0.0012	−0.0045

**Table 6:** Distance errors between global control points with angle optimization (unit: mm).

No.	$\Delta L_{12}$	$\Delta L_{13}$	$\Delta L_{14}$	$\Delta L_{23}$	$\Delta L_{24}$	$\Delta L_{34}$	Max Deviation	RMS Error
Before Optimization	0.0157	0.0179	0.0108	0.0018	0.0068	0.0032	0.0179	0.0111
After Optimization	0.0081	0.0115	0.0090	0.0005	0.007	0.0012	0.0115	0.0074

**Table 7:** Statistical results (unit: mm).

NO.	D1	D2	D3	D4
1	0.016	0.037	0.009	0.025
2	0.016	0.026	0.014	0.021
3	0.012	0.022	0.011	0.017
4	0.020	0.041	0.014	0.029
5	0.018	0.031	0.032	0.031
6	0.020	0.010	0.011	0.035
7	0.007	0.020	0.005	0.026
8	0.009	0.021	0.012	0.037
9	0.021	0.015	0.016	0.032
10	0.017	0.023	0.006	0.022
μ	0.0156	0.0246	0.0130	0.0275
σ	0.0048	0.0095	0.075	0.0064
RMS error	0.0162	0.0262	0.0148	0.0281
